# Evaluation of Four Rapid Antigen Tests for the Detection of SARS-CoV-2 Infection with Nasopharyngeal Swabs

**DOI:** 10.3390/biomedicines11030701

**Published:** 2023-02-24

**Authors:** Ho-Jae Lim, Min-Young Park, Young-Hyun Baek, Hyeon-Seo Lee, Inhee Kim, Youngjin Kwon, Youngshin You, Kyoungwoo Nam, Jae-Hyun Yang, Min-Jin Kim, Nae Yu, Yong-Hak Sohn, Jung-Eun Park, Yong-Jin Yang

**Affiliations:** 1 Department of Molecular Diagnostics, Seegene Medical Foundation, Seoul 04805, Republic of Korea; 2 Department of Integrative Biological Sciences & BK21 FOUR Educational Research Group for Age-Associated Disorder Control Technology, Chosun University, Gwangju 61452, Republic of Korea; 3 Paul F. Glenn Center for Biology of Aging Research, Department of Genetics, Blavatnik Institute, Harvard Medical School, Boston, MA 02115, USA

**Keywords:** SARS-CoV-2, rapid antigen test, rRT-PCR, cut off

## Abstract

Owing to the high transmissibility of severe acute respiratory syndrome coronavirus 2 (SARS-CoV-2) variants, the capacity of testing systems based on the gold standard real-time reverse transcription–polymerase chain reaction (rRT-PCR) is limited. Rapid antigen tests (RATs) can substantially contribute to the prevention of community transmission, but their further assessment is required. Here, using 1503 nasopharyngeal swabs, we compared the diagnostic performance of four RAT kits (Abbott Panbio™ COVID-19 Ag Rapid Test, SD Biosensor Standard™ Q COVID-19 Ag Test, Humasis COVID-19 Ag Test, and SG Medical Acrosis COVID-19 Ag Test) to the cycle threshold (Ct) values obtained from rRT-PCR. The precision values, area under the curve values, SARS-CoV-2 variant detection ability, and non-SARS-CoV-2 specificity of all four kits were similar. An assay using the Acrosis kit had a significantly better positive detection rate with a higher recall value and cut-off value than that using the other three RAT kits. During the current COVID-19 pandemic, the Acrosis kit is an effective tool to prevent the spread of SARS-CoV-2 in communities.

## 1. Introduction

The coronavirus disease 2019 (COVID-19) pandemic, caused by severe acute respiratory syndrome coronavirus 2 (SARS-CoV-2), started in December 2019, and it remains a global public health concern [1]. The COVID-19 pandemic has subsided to a seasonal illness through vaccination campaigns [2]. Despite stringent efforts to control the spread of SARS-CoV-2, more than 633 million confirmed cases and 6.6 million deaths had been reported as of 17 November 2022 [3]. The fight against SARS-CoV-2 is hampered by coinfection with other respiratory viruses, such as influenza virus, respiratory syncytial virus, and non-SARS-CoV-2 coronavirus [4].

SARS-CoV-2 has one of the largest RNA genomes (~29.9 kb) among all RNA viruses [5]. Owing to the high mutation rate of RNA viruses, SARS-CoV-2 continuously accumulates multiple mutations, resulting in numerous variants of concern by December 2020 [6]. These variants have shown increased transmissibility because of extensive mutations and available diagnostic tools have become less effective [7]. Currently, various diagnostic tests for SARS-CoV-2 include real-time reverse transcription–polymerase chain reaction (rRT-PCR)-based nucleic acid assays, immunoassays (e.g., lateral flow antigen test), and clinical imaging (e.g., computed tomography scan) [8].

The rRT-PCR assay is the gold-standard test for SARS-CoV-2 detection, especially when using nasopharyngeal swabs (NPSs) obtained from suspected cases by trained healthcare workers [9]. However, rRT-PCR assays have a turnaround time of approximately 24–48 h, which results in a bottleneck in PCR testing during the peak period of SARS-CoV-2 infections, which adversely affects the management of patients with COVID-19 [10]. Thus, there is a need for further studies to develop and evaluate more rapid and feasible assays, such as rapid lateral flow tests.

Unlike rRT-PCR, the rapid lateral flow test employs direct detection methods, is easy to perform (and does not need to be performed by healthcare workers), and yields results within 30 min [11]. However, rapid lateral flow tests for the detection of antigens are considered less sensitive and less specific than rRT-PCR [12]. To overcome these limitations, various signal-amplified methods have been used for the sensitive detection of antigens in rapid lateral flow tests, including chemical enhancement, surface-enhanced Raman scattering, and fluorescence, as well as photothermal, electrochemical, and magnetic reactions [13]. Numerous studies have evaluated the sensitivity and reliability of the commercially available rapid antigen test (RAT) kits for SARS-CoV-2 detection [11,14,15]. However, only a few studies have analyzed the specificity of RATs for other respiratory viruses and SARS-CoV-2 variants or compared the results with the cycle threshold (Ct) values obtained from rRT-PCR assays [12,16].

To date, studies have demonstrated the performance of well-known rRT-PCR kits for SARS-CoV-2 detection [17,18]. Therefore, in this study, we compared the diagnostic performance of four RAT kits, namely the ACROSIS COVID-19 Antigen kit (SG Medical, Seoul, Republic of Korea), STANDARD^TM^ Q COVID-19 Ag Test kit (SD BIOSENSOR Inc., Suwon, Republic of Korea), Panbio^TM^ COVID-19 Ag Rapid Test kit (Abbott Diagnostics, Chicago, IL, USA), and Humasis COVID-19 Ag Test kit (Humasis Co., Ltd., Gunpo, Republic of Korea) to the Ct values obtained from rRT-PCR. These RAT kits are considered to yield results more quickly than the rRT-PCR assay with comparable accuracy. Therefore, we compared the diagnostic performance of these RAT kits with that of rRT-PCR using large-scale NPS samples (≥1500).

## 2. Materials and Methods

### 2.1. Storage of Clinical Specimens

This study was approved by the Institutional Review Board of the Seegene Medical Foundation (SMF-IRB-2022-025 approved on 25 July 2022). The requirement for informed consent from the participants was waived because the samples collected for this study were anonymized. NPS specimens (*n* = 1503) were obtained during routine diagnostic testing conducted from December 2020 to October 2022 and evaluated using the SARS-CoV-2 molecular assay. The results of the analysis of cross-reactivity tests showed that 67 of the SARS-CoV-2-negative (*n* = 701) samples provided were positive for other known respiratory viruses. Briefly, the samples with Ct values < 26 were considered positive and classified under 14 species as follows: human coronaviruses OC43 (*n* = 5), NL63 (*n* = 4), and 229E (*n* = 3); influenza virus types A (*n* = 5) and B (*n* = 5); respiratory syncytial virus types A (*n* = 5) and B (*n* = 6); parainfluenza virus types 1 (*n* = 4) and 4 (*n* = 5); bocavirus (*n* = 5); enterovirus (*n* = 5); rhinovirus (*n* = 5); metapneumovirus (*n* = 5); and adenovirus (*n* = 5). The specimens were anonymized, aliquoted into 1.5 mL conical tubes, and stored at −70 °C until use (freeze/thaw cycles ≤ 1). Samples of sufficient volume (≥700 µL) for all four RATs, as well as for nucleic acid extraction before rRT-PCR, were preserved in a universal transport medium (UTM) without inactivation.

### 2.2. Nucleic Acid Extraction and rRT-PCR Analysis

The rRT-PCR assay was used as the gold standard; the assay was performed with purified nucleic acid and a commercially available kit using the Allplex^TM^ SARS-CoV-2 Assay (Seegene Inc., Seoul, Republic of Korea). The nucleic acid was extracted from 1503 post-thaw samples using the MagNA Pure 96 system (Roche Inc., Basel, Switzerland) as previously described [19]. The purified nucleic acids were then used in the rRT-PCR, which detects the RNA-dependent RNA polymerase (*RdRP*) and spike (*S*), envelope (*E*), and nucleocapsid (*N*) genes. The rRT-PCR assay with the Allplex^TM^ SARS-CoV-2 Assay kit was performed using CFX96^TM^ (Bio-Rad Laboratories, Hercules, CA, USA). The thermal cycling conditions were as follows: 50 °C for 20 min; 95 °C for 15 min; and 45 cycles at 95 °C for 10 s, 60 °C for 15 s, and 72 °C for 10 s. The data were analyzed using Seegene Viewer (v3.24; Seegene Inc.). The results were interpreted as SARS-CoV-2 positive when the Ct value was ≤40 for all targets, according to the manufacturer’s instructions [9]. Of these, *RdRP* and *S* were represented by a Ct value from rRT-PCR in this study. Positive samples were then categorized into three different groups based on their Ct value: Ct < 20, 20 ≤ Ct < 30, and Ct ≥ 30. Of these, the 20 ≤ Ct < 30 group was subdivided into five groups with Ct values of 20 ≤ Ct < 22, 22 ≤ Ct < 24, 24 ≤ Ct < 26, 26 ≤ Ct < 28, and 28 ≤ Ct < 30.

### 2.3. RAT Kits and Experimental Protocol

The 1503 thawed samples were tested simultaneously for the SARS-CoV-2 N protein using four commercially available RAT kits: (i) Panbio^TM^ COVID-19 Ag Rapid Test kit (Panbio^TM^), (ii) STANDARD^TM^ Q COVID-19 Ag Test kit (Standard^TM^), (iii) Humasis COVID-19 Ag Test kit (Humasis), and (iv) Acrosis COVID-19 Antigen kit (Acrosis). The standard procedure was modified according to a previously reported protocol [20,21]. Briefly, we mixed 60 μL of UTM with 60 μL of extraction buffer (1:1 ratio); this is not routinely applied in RAT protocols. The mixtures were transferred to their respective sample wells following the manufacturer’s recommendation and were maintained at 20–24 °C for an additional 15 min. The results were then analyzed by two authors (Y.H.B. and H.S.L.); a positive result was one in which a band was visible at the “T” site. In the event of discrepant interpretations, another author was consulted to make a final decision (H.J.L. or Y.Y.).

### 2.4. Next Generation Sequencing (NGS)

Using the RAT kit-tested samples, an NGS analysis was conducted below the minimum cut-off of the randomly selected SARS-CoV-2-positive samples using the MiSeq platform (Illumina, San Diego, CA, USA) following a previously published protocol [22]. Briefly, 55 ng of nucleic acid were processed using SARS-CoV-2 FLEX Panels (Paragon Genomics, Hayward, CA, USA). The sequence files were analyzed using the Flomics pipeline (Flomics, Barcelona, Spain) and were assigned to a sublineage by the Pangolin Tool (ver.4.1.3). Genetic mutations in *N* were identified by Nextclade (ver. 2.8.1).

### 2.5. Statistical Analysis

Rstudio (ver. 4.1.2; RStudio Inc., Boston, MA, USA) was used to perform all statistical analyses and generate graphs. The performance of each RAT kit was evaluated by estimating precision and recall against the rRT-PCR results using the caret package [23]. The correlation between the Ct values from rRT-PCR and the results of assays using each RAT kit was evaluated using the log-rank test in the survminer package [24]. The receiver operating characteristic (ROC) curve and area under curve (AUC) were generated using the pROC package, and the optimal cutoffs for each RAT were calculated using scores that estimated the minimum distance between the left-upper corner and ROC curve [25]. The dot plots were generated using the ggpubr package. The true-positive rate (proportion of RAT-positive results in SARS-CoV-2-positive samples) was used to compare the differences among the RAT kits. The results were considered statistically significant at *p* < 0.05.

## 3. Results

### 3.1. Comparison of Detection Rates of the RAT Kits

The clinical performance of the four RAT kits was compared using 1503 samples. Of the 1503 NPS samples, 802 were SARS-CoV-2 positive, as determined using rRT-PCR (Table 1). Without inhibiting the control line, all the RAT kits were effective for the UTM samples. Compared with the rRT-PCR assay, the assay using the RAT kits had a precision rate of 99.32–100%; however, the recall rate was 76.56% for Acrosis and between 53.87% and 55.74% for the other three kits (Table 1). Overall, the performance of the Acrosis kit was better than that of the other three kits for the SARS-CoV-2-positive samples.

### 3.2. Positivity and Cross-Reactivity of the RAT Kits

The 802 SARS-CoV-2-positive samples in the rRT-PCR analysis (Table 2) were classified based on their Ct value (Ct < 20: *n* = 137; 20 ≤ Ct < 30: *n* = 513; or 30 ≤ Ct < 40: *n* = 152). Of these, 513 samples (20 ≤ Ct < 30) were further classified as follows: 20 ≤ Ct < 22 (*n* = 110), 22 ≤ Ct < 24 (*n* = 96), 24 ≤ Ct < 26 (*n* = 100), 26 ≤ Ct < 28 (*n* = 102), and 28 ≤ Ct < 30 (*n* = 105).

The RAT results showed that 29.5% (444/1503), 29.8% (448/1503), 28.7% (432/1503), and 40.9% (614/1503) of the samples tested positive in the assays using the Panbio^TM^, Standard^TM^, Humasis, and Acrosis kits, respectively. Of the 701 samples that tested negative in the rRT-PCR analysis, three were positive (0.4%) in the assay using the Panbio^TM^ kit and one (0.1%) was positive in the assay using the Standard^TM^ kit (Table 2). We compared the cross-reactivity of RATs using samples infected with other respiratory viruses in the SARS-CoV-2-negative group. The cross-reactivity of 67 samples was 100% with no false positives for SARS-CoV-2 (Supplementary Table S1). Of the remaining 802 SARS-CoV-2-positive samples, two RAT kits (Panbio^TM^ and Humasis) reliably detected samples with a Ct value of ≤20 per kit. However, the other two RAT kits, Standard^TM^ and Acrosis, detected samples with values of 22 and 24 Ct, respectively. The Acrosis kit was reasonably sensitive, showing 100% SARS-CoV-2 detection in samples with a Ct value < 24 (Table 2). In addition, it showed no cross-reactivity for 14 species of non-SARS-CoV-2 pathogens (Supplementary Table S1). The log-rank test was used to compare the positive rate in the assays with the four kits (Table 3). There was no significant difference in the results obtained using the Standard^TM^, Panbio^TM^, and Humasis kits (*p* > 0.05). However, there was a significant difference in the positive rate in the assay using the Acrosis kit compared with the other three RAT kits (*p* < 0.001).

### 3.3. Optimal Cut-Off Values Based on Ct Values

**Figure 1:**
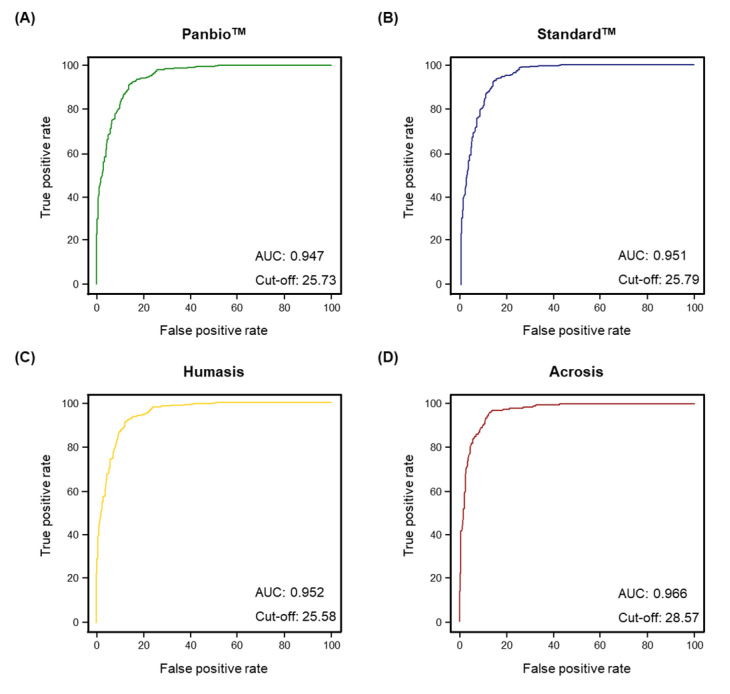
Performance of the four RAT kits in detecting SARS-CoV-2 (based on Ct values). ROC curves showing the diagnostic performance of the (**A**) Panbio^TM^, (**B**) Standard^TM^, (**C**) Humasis, and (**D**) Acrosis kits. The colored lines represent the diagnostic performance of each RAT kit.

The ROC curves were constructed to examine the diagnostic accuracy of the four RAT kits (Figure 1). The cut-off scores of the RAT kits are summarized using dot plots (Supplementary Figure S1). The AUCs of the Ct values, which were used to discriminate between RAT-positive and RAT-negative results, ranged from 0.947 to 0.966. The highest optimal cut-off score determined from the Acrosis kit was 28.57, whereas the cut-off scores for the other three kits ranged from 25.58 to 25.79. Thus, the cut-off value for the Acrosis kit was higher (by 2.78–2.99) than that for the other three kits.

### 3.4. True-Positive Rate of the RAT Kits for SARS-CoV-2 Variants below the Cut-Off Values

Among the 427 SARS-CoV-2-positive samples with Ct values below 25.58, 77 (18.0%) were analyzed using NGS (Table 4). The NGS analysis confirmed the three SARS-CoV-2 variants (pre-Delta, Delta, and Omicron). The SARS-CoV-2 variants were classified into 10 lineages as follows: 3 lineages of pre-Delta variants (8 samples for B.1.497, 3 for B.1.619, and 4 for B.1.620), 2 lineages of Delta variants (3 samples for AY.69 and 4 for AY.122.5), and 5 lineages of Omicron variants (2 samples for BA.1, 17 for BA.2, 1 for BA.4, 33 for BA.5, and 2 for BE.1). Of these, three to five samples had discrepant results in the analysis using the Panbio^TM^ kit (two samples for B.1.620 and two for BA.5), Standard^TM^ kit (one sample for B.1.620 and two for BA.5), and Humasis kit (two samples for B.1.620 and three for BA.5). These findings suggest that the Acrosis kit has a remarkable superiority in detecting the 10 lineages of the three SARS-CoV-2 variants. For social activity settings requiring a negative SARS-CoV-2 test, the Acrosis kit can serve as an effective tool for regular screening.

### 3.5. Additional Mutations in Samples of the B.1.619 and BA.5 Lineages with Discordant Results

To determine the genetic mutation site causing discordant results in the RAT kit assays, an NGS analysis was performed. A220F was a common mutation in the B.1.620 lineage, and P13L, ERS31–33del, R203K, G204R, and S413R were identified as common mutations in the BA.5 lineage (Table 5). In the discordant samples analyzed using the RAT kits (Table 4), L221F was observed to be the additional mutation in the B.1.620 lineage in two samples, whereas the BA.5 lineage had no additional mutation in three samples (Table 5). There were no differences in additional mutations between the concordant and discordant samples. These findings suggest that the discordant samples would not have affected *N* mutations.

## 4. Discussion

SARS-CoV-2 is the third most highly pathogenic human coronavirus next to SARS-CoV and MERS-CoV and is rapidly transmitted from infected individuals [26]. SARS-CoV-2 accumulates genetic mutations, resulting in the emergence of new variants, such as pre-Delta (e.g., B.1.1.7, B.1.351, B.1.497, and B.1.619), Delta (e.g., B.1.617.2, AY.69, and AY.122.5), and Omicron variants (e.g., BA.1, BA.2, and BA.3) [27]. Newly emerging variants are rapidly evolving, thereby increasing both the number of confirmed cases and new mutations, which can affect the sensitivity of diagnostic tests [28]. Therefore, a rapid and accurate diagnostic test for SARS-CoV-2 infection is crucial for the effective prevention of disease transmission.

The RAT kits are simple and reliable on-site testing tools used for the rapid (less than 30 min) detection of SARS-CoV-2 infection from self-collected nasal swabs [11]. A previous study reported a similar performance between self-collected and healthcare-worker-collected samples [9]. The self-collection of nasal and oropharyngeal swabs is promoted to screen for widespread infection without delay [29,30]. It was an effective approach during the COVID-19 pandemic as it reduced the community transmission of SARS-CoV-2 [9]. Despite these advantages, numerous studies have reported a lower analytical sensitivity of RAT kits than that of rRT-PCR methods and analyzed a wide range of Ct values in the optimal cut-off score [31,32,33]. Thus, in this study, the cut-off values from Ct values between 20 and 30 were estimated using a similar number of samples.

The aim of this comparative study was to evaluate the performance of four commercial RAT kits to detect SARS-CoV-2 infection in NPS samples using the established rRT-PCR as a reference test. None of the RATs displayed cross-reactivity with 14 other respiratory viruses (Table S1), and all the tests had similar AUC values (Figure 1). However, three RAT kits (Panbio^TM^, Standard^TM^, and Humasis) showed divergent results for two lineages (among five samples) unlike the Acrosis kit (Table 4). The *N* mutations (P13L, ERS31–33del, A182T, R203K, G204R, A220F, L221F, and S413R) found in this study did not correlate between the consistent and inconsistent samples (Table 5). These findings indicate that these RATs are highly specific for SARS-CoV-2.

It has been reported that the screening of asymptomatic patients using RAT kits is limited [34] and the sensitivity of RAT kits reduces the number of true-positive results with Ct values > 25 [35,36]. The primary finding of this study was that the Acrosis kit showed a high recall rate (Table 1) and highly sensitive optimal cut-off Ct value of 28.57 (Supplementary Figure S1D) compared with the other three RAT kits (Supplementary Figure S1A–C). In terms of rRT-PCR Ct values, the performance of the Acrosis kit was significantly better than that of the other three kits (*p* < 0.001; Table 3). Notably, the Acrosis kit showed 6.87–7.94 fold (3 Ct) more sensitivity than the other three kits (Figure 1). Therefore, the Acrosis kit can be an effective screening tool for detecting infected individuals and protecting the community.

This study has two major limitations. First, the test kit manufacturers recommended the use of NPS specimens; these swabs should be placed in the provided extraction buffer. However, in this study, the UTM was mixed with the provided extraction buffer (at a ratio of 1:1). Second, the discrepancies identified in five samples could not be understood. Furthermore, the NPS samples were freeze thawed under identical sample conditions; however, the effect of the freeze-thawed samples on RATs has not been evaluated. Therefore, further studies to evaluate the RAT kits using fresh, unfrozen nasal swabs for the detection of SARS-CoV-2 infection are required.

## 5. Conclusions

Owing to the emergence of multiple, highly transmissible SARS-CoV-2 variants since December 2020, alternative testing methods with short turn-around times are required to prevent community virus transmission. In this study, we obtained three major findings. First, the true-positive rate of the Acrosis kit was significantly higher than that of the other three kits. Second, all RAT kits evaluated in this study can detect 10 lineages of SARS-CoV-2 variants (including Pre-Delta, Delta, and Omicron), despite differences in the cut-off value. Third, none of the RAT kits detected 14 other respiratory pathogens, indicating high specificity to SARS-CoV-2. Our results indicate that the Acrosis kit can be used in daily clinical practice for SARS-CoV-2 detection; however, additional studies on the field performance of the kit are required to determine its efficiency.

## Figures and Tables

**Table 1:** Comparison of the diagnosis performance of the RAT kits.

RAT	rRT-PCR					
	TP (*n*)	FP (*n*)	TN (*n*)	FN (*n*)	Precision (%)	Recall (%)
Panbio^TM^	441	3	698	361	99.32	54.99
Standard^TM^	447	1	700	355	99.78	55.74
Humasis	432	0	701	370	100	53.87
Acrosis	614	0	701	188	100	76.56

Data indicate the number of samples or percentages. TP, true positive; FP, false positive; TN, true negative; FN, false negative.

**Table 2:** RAT results stratified using a detailed classification based on the Ct value.

RAT	rRT-PCR								
	Total	Neg.	Ct < 20	20 ≤ Ct < 22	22 ≤ Ct < 24	24 ≤ Ct < 26	26 ≤ Ct < 28	28 ≤ Ct < 30	Ct ≥ 30
Panbio^TM^	29.5% (444/1503)	0.4% (3/701)	100% (137/137)	98.2% (108/110)	89.6% (86/96)	60.0% (60/100)	29.4% (30/102)	14.3% (15/105)	3.3% (5/152)
Standard^TM^	29.8% (448/1503)	0.1% (1/701)	100% (137/137)	100% (110/110)	92.7% (89/96)	61.0% (61/100)	28.4% (29/102)	12.4% (13/105)	5.3% (8/152)
Humasis	28.7% (432/1503)	0% (0/701)	100% (137/137)	98.2% (108/110)	89.6% (86/96)	59.0% (59/100)	23.5% (24/102)	12.4% (13/105)	3.3% (5/152)
Acrosis	40.9% (614/1503)	0% (0/701)	100% (137/137)	100% (110/110)	100% (96/96)	97.0% (97/100)	95.1% (97/102)	55.2% (58/105)	12.5% (19/152)

Data are presented as percentage (number of RAT-positive samples/rRT-PCR-positive samples). Neg., negative.

**Table 3:** Comparison of the true-positive results for RAT kits.

Comparison Kits	*N*	Difference	*p*
Panbio^TM^ vs. Standard^TM^	1503	–4	0.82
Panbio^TM^ vs. Humasis	1503	12	0.74
Panbio^TM^ vs. Acrosis	1503	–170	<0.001 *
Standard^TM^ vs. Humasis	1503	16	0.58
Standard^TM^ vs. Acrosis	1503	–166	<0.001 *
Humasis vs. Acrosis	1503	–182	<0.001 *

The *p*-value represents the difference between the two curves according to the log-rank test results. * *p* < 0.05.

**Table 4:** True-positive values of the RAT kits according to the SARS-CoV-2 lineages.

Variants	Lineage	*N*	Rapid Antigen Test			
			Panbio^TM^	Standard^TM^	Humasis	Acrosis
Pre-Delta	B.1.497	8	8/8	8/8	8/8	8/8
	B.1.619	3	3/3	3/3	3/3	3/3
	B.1.620	4	2/4 *	3/4 *	2/4 *	4/4
Delta	AY.69	3	3/3	3/3	3/3	3/3
	AY.122.5	4	4/4	4/4	4/4	4/4
Omicron	BA.1	2	2/2	2/2	2/2	2/2
	BA.2	17	17/17	17/17	17/17	17/17
	BA.4	1	1/1	1/1	1/1	1/1
	BA.5	33	31/33 *	31/33 *	30/33 *	33/33
	BE.1	2	2/2	2/2	2/2	2/2

Data indicate the number of RAT-positive/rRT-PCR-positive samples. * False-negative result was observed in SARS-CoV-2-positive samples.

**Table 5:** Comparison of *N* mutations in the B.1.620 and BA.5 lineages.

Lineage	*N*	Result	Ct Value	Mutation	
				Common	Additional
B.1.620	1	Con.	17.07	A220F	-
	1	Con.	22.55		L221F
	2	Dis.	21.34, 22.19		L221F
BA.5	28	Con.	16.06–24.88	ERS31–33del, P13L, R203K, G204R, S413R	-
	3	Dis.	18.63–24.40		-
	2	Con.	22.99, 23.37		A182T

Data indicate the number of concordant and discordant samples with the corresponding mutations. Con., concordant; Dis., discordant.

## Data Availability

All data are available within the article.

## Supplementary Materials

The following are available online at https://www.mdpi.com/article/10.3390/biomedicines11030701/s1: Supplementary Table S1: Cross-reactivity of the RAT kits with other respiratory pathogens; Supplementary Figure S1: SARS-CoV-2-positivity rate according to the RAT kits (based on the Ct values).
