# Supplementary material for: Evaluation of Four Rapid Antigen Tests for the Detection of SARS-CoV-2 Infection with Nasopharyngeal Swabs
Source: Biomedicines. 2023 Feb 24;11(3):701. doi: 10.3390/biomedicines11030701 (PMC10045780; doi:10.3390/biomedicines11030701)
Supplement: Supplementary file 1 [file biomedicines-11-00701-s001.zip › biomedicines-2196227-supplementary.pdf]

Supplementary Table S1. Cross-reactivity of the RAT kits with other respiratory pathogens

Virus	*N* of samples	Rapid antigen test			
		**Panbio™**	**Standard™**	**Humasis**	**Acrosis**
HCoV-OC43	5	0/5	0/5	0/5	0/5
HCoV-NL63	4	0/4	0/4	0/4	0/4
HCoV-229E	3	0/3	0/3	0/3	0/3
Influenza virus A	5	0/5	0/5	0/5	0/5
Influenza virus B	5	0/5	0/5	0/5	0/5
RSV type A	5	0/5	0/5	0/5	0/5
RSV type B	6	0/6	0/6	0/6	0/6
PIV type 1	4	0/4	0/4	0/4	0/4
PIV type 4	5	0/5	0/5	0/5	0/5
Adenovirus	5	0/5	0/5	0/5	0/5
Metapneumovirus	5	0/5	0/5	0/5	0/5
Rhinovirus	5	0/5	0/5	0/5	0/5
Enterovirus	5	0/5	0/5	0/5	0/5
Bocavirus	5	0/5	0/5	0/5	0/5

Data indicate the number of RAT-positive samples / rRT-PCR-positive samples. HCoV, human coronavirus; RSV, respiratory syncytial virus; PIV, parainfluenza virus.

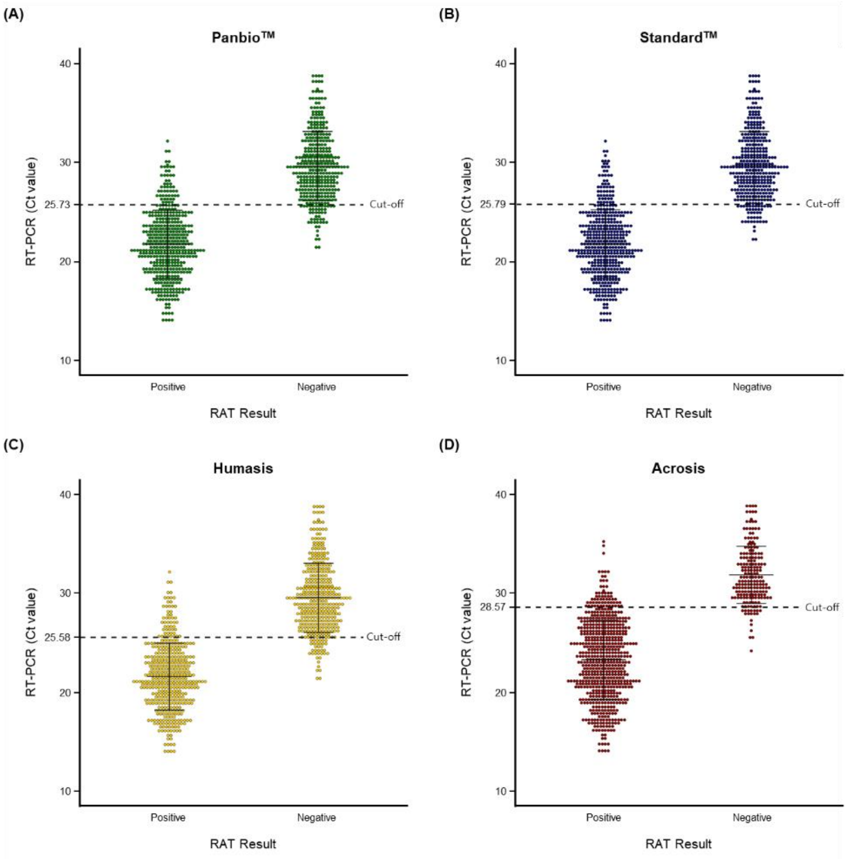

Supplementary Figure S1. SARS-CoV-2-positivity rate according to the RAT kits (based on the Ct values). Dot plots showing the positive and negative samples detected using the (A) Panbio™, (B) Standard™, (C) Humasis, and (D) Acrosis kits. The colored circles represent the RAT results of individual samples. The dotted lines represent the cut-off values for each RAT kit.
